# Safety and Long-Term Survival Outcome in Patients With Unresectable Barcelona Clinic Liver Cancer (BCLC) Stages C and D Advanced Hepatocellular Carcinoma Treated With 40 μm Drug-Eluting Bead Transcatheter Arterial Chemoembolization

**DOI:** 10.7759/cureus.24047

**Published:** 2022-04-11

**Authors:** Michael Wholey, Raul Palacios III, Daniel Wholey, Alejandro Mendez

**Affiliations:** 1 Interventional Radiology, University of Texas Health San Antonio, San Antonio, USA; 2 Department of Radiology, Audie Murphy Veterans Administration Hospital, San Antonio, USA; 3 Department of Statistics, Boston College, Chestnut Hill, MA, USA

**Keywords:** doxorubicin-loaded microspheres, barcelona clinic liver cancer stage, microspheres, hepatocellular carcinoma, deb-tace

## Abstract

Background

To evaluate the safety, treatment response, and overall survival (OS) following drug-eluting bead transarterial chemoembolization (DEB-TACE) using doxorubicin-loaded 40 μm microspheres in patients with advanced hepatocellular carcinoma (HCC).

Methods

This was a single-center retrospective evaluation of patients with unresectable HCC without extrahepatic spread and Barcelona Clinic Liver Cancer (BCLC) stages C and D disease who underwent DEB-TACE between August 2015 and January 2018. Pre-treatment data included demographics, medical history, cancer staging, tumor size, laboratory results, and prior treatments for HCC. Follow-up data included the date of DEB-TACE treatments or microwave ablation (MWA) procedures, laboratory test results, adverse events, treatment response, and the date and cause of death.

Results

Thirty-two patients met the study inclusion criteria. Eighteen patients (56.3%) underwent a single DEB-TACE and 14 patients (43.8%) had two to five DEB-TACE procedures. Five patients (15.6%) had MWA following initial DEB-TACE. Mild postembolization syndrome occurred in six patients (18.8%) during a 30-day period following initial DEB-TACE. Seven patients (21.9%) experienced worsening ascites, pleural fluid, or encephalopathy during the study observation period. Three patients had moderate to severe worsening liver function tests 90 days post-procedure. Seventeen patients (53.1%) had a complete or partial response and nine patients (28.1%) had disease progression. Median OS was 15.0±14.4 months from the time of initial DEB-TACE, with 63% and 33% of patients still alive at 12 and 24 months. Multivariate analyses identified that Okuda Stage (P=0.03) and Cancer of the Liver Italian Programme (CLIP) score (P=0.05) were significantly associated with overall survival after adjusting for other covariates. There were four HCC-related deaths during the 30-day post-procedure period.

Conclusion

DEB-TACE with small 40 μm doxorubicin-loaded microspheres is a safe and effective treatment for unresectable patients with BCLC stages C and D advanced HCC. Patients with advanced, high-risk unresectable HCC should be considered for DEB-TACE as opposed to supportive or palliative care alone.

## Introduction

Liver cancer was the sixth most common cancer worldwide and the third most common cause of cancer death in 2020 [1]. There were an estimated 42,000 new cases of liver and intrahepatic bile duct cancer in the United States in 2020 and approximately 30,000 deaths [2]. Hepatocellular carcinoma (HCC) is the most common type of liver cancer, occurring in approximately 75% to 85% of all liver cancer patients and increasing at a rate of 2% to 3% annually [2-3].

While several staging systems for HCC exist, the Barcelona Clinic Liver Cancer (BCLC) staging and prognostic system is widely accepted in clinical practice and endorsed by the American Association for the Study of Liver Diseases (AASLD) and the European Association for the Study of the Liver - European Organisation for Research and Treatment of Cancer (EASL-EORTC) since it incorporates tumor size, liver function, and Eastern Cooperative Oncology Group (ECOG) performance status [4-6]. While curative treatment options, such as surgical resection, liver transplantation, and ablation are recommended for patients with early-stage cancer (BCLC stages A and B), patients with BCLC stages C and D liver cancer have reduced treatment options and a poorer prognosis [4].

As a result of limited treatment options for patients with unresectable advanced stage HCC, the identification of safe and effective treatment options is needed. Transarterial chemoembolization (TACE) is an established therapy for treating unresectable HCC [7]. Currently, the use of drug-eluting bead transarterial chemoembolization (DEB-TACE) has become a standard technique for the treatment of unresectable HCC. The calibrated microspheres, typically loaded with doxorubicin, enable sustained and selective delivery of the drug to the tumor [8]. Compared to TACE, DEB-TACE enables both the embolization of arterial vessels supplying the tumor and the more local delivery of high levels of chemotherapy within the tumor [9]. The reduced requirement for systemic drug delivery with DEB-TACE compared to TACE has been reported to be associated with a decrease in post-treatment toxicity [10].

While the standard diameter for DEB is typically 100 to 300 μm, a smaller size may permit the placement of beads more proximally and closer to the tumor, and the delivery of more localized drug therapy with a decreased risk of systemic adverse events [11]. Several studies have reported the use of DEB with a diameter of less than 100 μm for the treatment of unresectable HCC in patients who were not eligible for ablation or surgery [12-14]. The authors of these studies indicated that the use of smaller beads resulted in a lower plasma maximum and area under the curve (AUC) for doxorubicin [12], higher response rates and survival duration as compared to larger beads [13], and a good safety profile [14].

As a result of the above findings and the poor outcomes associated with patients with advanced HCC, the aim of the present study was to retrospectively evaluate outcomes associated with the use of doxorubicin-loaded 40 μm microspheres for DEB-TACE in patients classified as having BCLC stages C and D liver cancer in a real-world setting. The primary objective of the study was to evaluate the safety of the use of these smaller beads in this patient population. Secondary objectives included the assessment of treatment response and patient survival following DEB-TACE treatment and the impact of differing lines of treatment following initial DEB-TACE with the 40 μm microspheres.

## Materials and methods

The study retrospectively analyzed the medical records of patients with advanced HCC who received DEB-TACE between August 2015 and January 2018. Inclusion criteria were patients with unresectable advanced HCC (BCLC stages C and Stage D) who underwent DEB-TACE with doxorubicin-loaded 40 μm Oncozene® Microspheres (Varian Medical Systems, Palo Alto, CA). Exclusion criteria were evidence of metastatic spread, a total bilirubin >3.0, and an international normalized ratio (INR) >2.0. Patient outcomes were assessed until April 2020 or death if it occurred prior to this date. The study received institutional review board (IRB) approval from the University of Texas Health Science Center at San Antonio and the Audie Murphy VA Hospital in San Antonio, with both IRBs waiving the need for informed consent from patients. The methods used for the study were carried out in accordance with relevant guidelines and regulations.

Patient characteristics

Patients’ baseline demographic and clinical characteristics were obtained from electronic medical records. This included age, sex, liver disease-related comorbidities, BCLC staging [4], Eastern Cooperative Oncology Group (ECOG) performance status, Child-Pugh class [15-16], model for end-stage liver disease (MELD) score [17], Okuda staging [18], Cancer of the Liver Italian Programme (CLIP) score [19], tumor satisfying Milan criteria (MILAN) [20], previous DEB-TACE, ablation or sorafenib treatment, number and location of lesions treated with DEB-TACE, and periprocedural laboratory results.

DEB-TACE procedure

A 2 mL syringe of the 40 μm microspheres loaded with 75 mg of doxorubicin was prepared by the pharmacy. The contents of the 2 mL syringe were mixed with 20-30 cc of contrast media and 10-20 cc of saline in the procedure room and attached to a 3 ml or 5 ml syringe for administration. Care was taken to ensure no heparin was added to the saline since this interferes with the delivery of the doxorubicin. Patients were intravenously premedicated with 25 to 50 mg of diphenhydramine and 500 mg of levofloxacin, or an alternative if allergic. Moderate sedation with midazolam and fentanyl was used for the majority of the procedures.

The femoral artery was utilized as access to the vasculature. The hepatic artery was typically accessed using a 5 Fr Cobra, Reuter, or another angled catheter. After access was obtained to the common or proper hepatic artery, a 2.8 Fr microcatheter was used to sub-select arterial branches to the tumor. Once the most proximal vessel to the tumor was accessed, the beads were slowly injected until hemostasis was achieved. The DEB-TACE procedure was typically performed on an outpatient basis with the patient discharged the day of the procedure.

Follow-up assessment

All patients were followed up with either liver CT or MRI at six weeks, followed by a clinic visit. Follow-up imaging was then performed at three- and six-month intervals. Adverse events were reviewed according to the National Cancer Institute’s Common Terminology Criteria for Adverse Events (CTCAE) version 5 [21]. If there was an increase over baseline of the CTCAE grade of a toxicity post-DEB-TACE, the grade to which the toxicity increased was recorded. Hepatotoxicity during the first 90 days post-DEB-TACE was assessed according to the Drug-Induced Liver Injury Network (DILIN) [22]. Symptoms of the post-embolization syndrome and other toxicities were conservatively attributed to DEB-TACE even in cases of possible attribution to tumor progression, or cirrhosis. Acute toxicities were defined as any toxicity occurring within 90 days of DEB-TACE treatment.

The modified Response Evaluation Criteria in Solid Tumours (mRECIST), based on CT and MRI imaging results, was used to assess the treatment effect following DEB-TACE for primary targeted tumors and or subsequent treatments [23]. Patient response to treatment was defined as either a complete response (CR), partial response (PR), stable disease (SD), or disease progression. Overall survival (OS) was calculated from the time of treatment to the last follow-up date or patient death.

Statistical analysis

Statistical analyses for quantitative data associated with patient characteristics were run using Microsoft® Excel® for Microsoft 365 MSO (16.0.13029.20232) (Microsoft Corporation, Redmond, WA). Results included frequency counts and percentages, means, standard deviation (SD), and minimum and maximum. Categorical variables were summarized by frequencies and percentages. Statistical analyses for outcomes data were performed using SAS version 9.4 (SAS Institute, Cary, NC). Cox proportional hazards regression was performed for multivariate analysis of prognostic factors. Survival curves were constructed via the Kaplan-Meier method and comparisons were made using the log-rank test. Survival time was calculated from the date of first DEB-TACE treatment using doxorubicin-loaded 40 μm microspheres to the date of death due to any cause or censored on the date of the last contact for those still alive at the last follow-up. A two-sided p-value of <0.05 was considered statistically meaningful. As patients with the shortest survival do not live sufficiently long to receive more treatments, survival comparisons by treatment received are inherently biased in favor of patients with longer survival and those who received more treatments. To mitigate this bias, a landmark analysis was performed to compare patients receiving a single DEB-TACE treatment using doxorubicin-loaded 40 μm microspheres versus those with additional MWA treatment or multiple DEB-TACE treatments. Patients surviving less than 150 days past the first DEB-TACE treatment were excluded from the analysis, where 150 days was the mean time between treatments for patients receiving multiple DEB-TACE treatments.

## Results

Patient population

Thirty-six patients met the inclusion criteria with 32 patients included in the final analysis as a result of four patients being excluded due to having documented extensive tumor spread, lymph node involvement, or total bilirubin greater than 3.0. Baseline patient characteristics are shown in Table 1. The mean patient age was 65.5±7.5 years (range 52-90). All patients included in the study were male, which is in line with the patient population in a VA hospital setting. All patients included in the analysis had a history of either alcohol or hepatitis-related cirrhosis, with 13 patients (40.6%) having both conditions. Sixteen patients (50%) had an alpha-fetoprotein (AFP) less than 25 ng/ml, 11 patients (34.4%) had an AFP between 26 and 3000 ng/mL, and five patients (15.6%) had an AFP greater than 3000 ng/mL on the day of the procedure.

**Table 1:** Patient demographics and baseline characteristics Notes: *reported as mean ± SD; **Includes one patient who also received sorafenib Abbreviations: AFP, alpha-fetoprotein; ALT, alanine transaminase; AST, aspartate aminotransferase; INR, international normalized ratio; MWA, microwave ablation; TACE, transarterial chemoembolization

Demographic or Characteristic	
Mean age ± SD (range)	65.5±7.5 (52-90)
Male sex	32 (100%)
Ethnicity	
Ethnicity - Non-Hispanic	17 (53.1%)
Ethnicity - Hispanic	15 (46.9%)
Liver disease etiology	
Hepatitis	24 (75.0%)
Alcohol	20 (62.5%)
Non-alcoholic fatty liver disease	1 (3.1%)
Other liver disease-related pathologies	
Portal hypertension	24 (75.0%)
Varices	17 (62.5%)
Encephalopathy	15 (46.9%)
Ascites	8 (25.0%)
Laboratory testing*	
Serum albumin, g/dL	1.5 ± 0.8
Total bilirubin, mg/dL	3.2 ± 0.5
INR	1.3 ± 0.2
AST (units/L)	75.6 ± 60.9
ALT (unit/L)	49.2 ± 38.1
Creatinine (mg/dl)	1.0 ± 0.5
AFP (ng/mL)	622.3 ± 1083.7
Tumor size (cm)*	
All tumors	4.1 ± 2.9
Primary tumors	4.6 ± 3.1
Secondary tumors (n=13)	2.4 ± 1.2
Previous treatments	
TACE only**	5 (15.6%)
MWA only	5 (15.6%)
TACE + MWA**	4 (12.5%)

Fourteen patients (43.8%) had prior treatment for HCC. Three patients (9.4%) received DEB-TACE alone, five patients (15.6%) microwave ablation (MWA) alone, and four patients (12.5%) received both DEB-TACE and MWA. Two patients (6.3%) received prior treatment with sorafenib, with one also receiving DEB-TACE and the other having DEB-TACE and MWA. Previous DEB-TACE was performed using 100-300 μm microspheres (LC Bead™, Boston Scientific, Natick, MA).

Table 2 shows the classification of patients included in the study by differing HCC staging systems. Nine patients (28.1%) were classified as BCLC stage C and the remaining 23 patients (71.9%) were BCLC stage D. The mean Child-Pugh score was 10.4±1.5 (range 7 to 13) and the mean MELD score was 9.5±3.6 (range 5 to 18). Twelve patients (37.5%) had a CLIP score of less than 3 while 19 patients (59.4%) had a CLIP score of 3 or more. Eighteen patients (56.3%) were Okuda stage I and 13 patients (40.6%) were Okuda stage II or III. The Okuda stage and CLIP score were not available for one patient.

**Table 2:** Hepatocellular carcinoma staging Abbreviations: BCLC, Barcelona-Clinic Liver Cancer; ECOG, Status Eastern Cooperative Oncology Group; MELD: Model for End-Stage Liver Disease; CLIP, Cancer of the Liver Italian Program

	All patients (n=32)	BCLC Stage C (n=9)	BCLC Stage D (n=23)
ECOG performance status			
0	4 (12.5%)	1 (11.1%)	3 (13.0%)
1	18 (56.3%)	7 (77.7%)	11 (47.8%)
2	7 (21.9%)	0 (0.0%)	7 (30.4%)
3	3 (9.4%)	1 (11.1%)	2 (8.7%)
Child-Pugh			
B	8 (25.0%)	1 (11.1%)	7 (30.4%)
C	24 (75.0%)	8 (88.9%)	16 (69.6%)
MELD score			
1 to 5	1 (3.1%)	1 (11.1%)	0 (0.0%)
6 to 10	20 (62.5%)	4 (44.4%)	0 (0.0%)
11 to 15	7 (21.9%)	2 (22.2%)	16 (69.6%)
>15	3 (9.4%)	1 (11.1%)	5 (21.7%)
Missing	1 (3.1%)	1 (11.1%)	2 (8.7%)
Okuda stage			
I (no factors present)	18 (56.3%)	5 (55.5%)	13 (56.5%)
II (1 to 2 factors present)	12 (37.5%)	3 (33.3%)	9 (39.1%)
III (3 to 4 factors present)	1 (3.1%)	0 (0.0%)	1 (4.3%)
Missing	1 (3.1%)	1 (11.1%)	0 (0.0%)
CLIP score			
1	1 (3.1%)	1 (11.1%)	0 (0.0%)
2	11 (34.4%)	2 (22.2%)	16 (69.6%)
3	15 (46.8%)	5 (55.5%)	5 (21.7%)
4 or more	4 (12.5%)	0 (0.0%)	2 (8.7%)
Missing	1 (3.1%)	1 (11.1%)	
Milan criteria			
Yes	16 (50.0%)	3 (33.3%)	13 (56.5%)
No	15 (46.9%)	5 (55.5%)	10 (43.5%)
Missing	1 (3.1%)	1 (11.1%)	0 (0.0%)

Twenty-one patients (65.6%) had a single tumor, nine patients (28.1%) had two tumors, and two patients (6.3%) had three tumors. The size of the primary tumor was less than 3 cm for 10 patients (31.3%), between 3 and 5 cm for 15 patients (46.9%), and greater than 5 cm for seven patients (21.9%). Three patients (9.4%) had primary tumors larger than 10 cm. Only three of the secondary tumors (9.3%) were greater than 3 cm with the largest being 4.2 cm. A total of 45 tumors were treated with the 40 μm doxorubicin-loaded microspheres.

DEB-TACE with 40 µm microspheres and subsequent procedures

All 32 patients included in the present analysis were successfully treated with DEB-TACE. The mean time from diagnosis for HCC to the initial procedure with 40 μm microspheres was 15.1±22.0 months. Thirty patients (93.4%) were discharged on the same day of the procedure with antibiotics and pain medication.

Patients in the study underwent a mean of 1.66±0.92 DEB-TACE procedures. This included 18 patients (56.3%) undergoing a single DEB-TACE procedure, nine patients (28.1%) undergoing two procedures, four patients (12.5%) undergoing three procedures, and one patient (3.1%) undergoing five procedures. The mean time between subsequent DEB-TACE procedures was 4.9±4.6 months. All DEB-TACE procedures utilized the 40 μm microspheres with doxorubicin.

Five patients (15.6%) patients underwent MWA procedures after initial DEB-TACE treatment with 40 μm microspheres. The mean time between the initial DEB-TACE procedure and MWA was 5.9±2.6 months. MWA was performed in three patients who received a single DEB-TACE treatment and one patient each who received two DEB-TACE treatments. Six patients (18.8%) were referred to an outside institution for Yttrium-90 (Y-90) treatment, with five patients subsequently undergoing DEB-TACE treatment and the remaining patient dying before the treatment could be performed. Three patients (9.4%) were referred for transplant evaluation with one patient receiving a liver transplant 17.1 months after their initial DEB-TACE procedure with the 40 μm microspheres. This same patient also underwent a second DEB-TACE with the 40 μm microspheres and an MWA procedure at 14.4 and 10.6 months, respectively, prior to undergoing the liver transplant procedure.

Adverse events

Adverse events were assessed following the 32 initial DEB-TACE procedures and the 21 subsequent procedures for patients who underwent DEB-TACE on one or more occasions following the initial treatment. Clinical adverse events were identified for both the immediate 30 days following DEB-TACE and for the duration of the study (Table 3). No serious clinical adverse events above CTCAE grade 3 were identified. Seven patients (21.9%) experienced adverse events following initial DEB-TACE with six patients (18.8%) developing mild postembolization syndrome characterized by abdominal pain and fatigue and one patient (3.1%) experiencing a worsening of ascites. No patients experienced symptoms of postembolization syndrome after the 30-day period following initial DEB-TACE treatment. The five patients who developed worsening ascites or worsening pleural fluid had an onset of symptoms between one and 30 months (median 7 months) following their last DEB-TACE procedure.

**Table 3:** Clinical adverse events* *Based on the National Cancer Institute’s Common Terminology Criteria for Adverse Events (CTCAE) version 5 [21]

	During 30 day period following DEB-TACE	After the initial 30 day period following DEB-TACE
Postembolization syndrome	6	0
Worsening ascites	1	2
Worsening pleural fluid requiring a chest tube	0	2
Gastrointestinal bleed	0	4
Encephalopathy	0	2
Abscess	0	1
Biloma	0	1

Laboratory adverse events were characterized by an increase from baseline at 90 days using the DILI severity index [22]. Sixteen patients (50%) were characterized as having no change in liver function, 13 patients (40.6%) were classified as having a mild drug-induced injury, and one patient each was identified as having moderate, moderate to severe, and severe drug-induced injury. 

Treatment response

Table 4 shows tumor response for the patient population and by BCLC stage. Tumor response was evaluated using dynamic CT or MRI and mRECIST criteria [15]. A complete response was characterized by the disappearance of intratumoral arterial enhancement in all target lesions. A partial response was noted when there was at least a 30% decrease in the sum of diameters of viable target lesions from baseline and progressive disease when there was a 20% or greater increase in the sum of the diameters of viable target lesions since baseline. Stable disease was noted for any cases that were not classified as having either a partial response or progressive disease. Objective treatment response was greater in patients with BCLC stage C versus stage D HCC (77.8% vs. 43.4%) and no patients with stage C experienced progressive disease compared to 39.3% of patients with BCLC stage D HCC. The median time to best response was 1.5±2.7 months (range 0.9 to 7.9 months) following initial DEB-TACE.

**Table 4:** Primary tumor response to treatment based on mRECIST criteria Source: [23]

	All patients (n=32)	BCLC stage C (n=9)	BDLC stage D (n=23)
Complete response	5 (15.6%)	2 (22.2%)	3 (13.0%)
Partial response	12 (37.5%)	5 (55.5%)	7 (30.4%)
Stable disease	6 (18.8%)	2 (22.2%)	4 (17.4%)
Progressive disease	9 (28.1%)	0 (0%)	9 (39.1%)
Objective response	17 (53.1%)	7 (77.8%)	10 (43.4%)
Clinical benefit rate	23 (71.9%)	9 (100%)	14 (60.9%)

Survival

Five patients (15.6%) were still alive at the end of the observation period. There were four deaths (12.5%) during the 30-day period following the initial DEB-TACE. Three of these deaths appeared to be related to cirrhosis and possibly a large tumor burden with tumor diameters for these patients being 9, 12, and 13 cm, respectively. The fourth death was related to a fall with the patient also having biochemical liver toxicity. Table 5 lists the causes of deaths related and unrelated to HCC with the majority of deaths (84.6%) due to HCC.

**Table 5:** Causes of deaths

Death related to HCC (n=22)	
Growth or spread of tumor in liver	6 (27%)
Cirrhosis-related variceal bleeding	3 (14%)
Cirrhosis hospice related	3 (14%)
Portal vein invasion and associated complications	2 (9%)
Severe ascites and pleural fluid	2 (9%)
Subdural hematoma after fall	2 (9%)
Pneumonia/acute respiratory distress syndrome (ARDS)	1 (5%)
End-stage renal disease	1 (5%)
Metastases to lymph nodes and adrenal glands	1 (5%)
Metastases to lung and bone	1 (5%)
Death unrelated to HCC (n=4)	
Cerebrovascular accident	1 (25%)
Urinary tract infection/sepsis	1 (25%)
Bladder cancer	1 (25%)
Leukemia	1 (25%)

**Figure 1:**
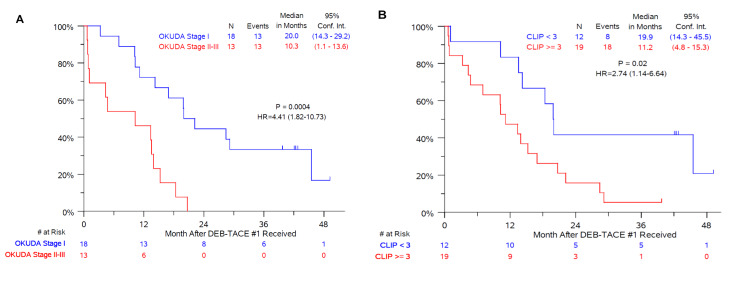
Kaplan-Meier curves showing overall survival following the first DEB-TACE treatment with 40 μm drug-eluting microspheres loaded with 75 mg of doxorubicin (A) Survival curve for patients classified as Okuda stage II or III versus Okuda I. (B) Survival curve for patients with a CLIP score greater than or equal to 3 versus less than 3. Okuda stage and CLIP score was not available for one patient in the series. Abbreviations: DEB-TACE: drug-eluting bead transarterial chemoembolization; CLIP: Cancer of the Liver Italian Programme

Median OS was 15.0±14.4 months from the time of the initial DEB-TACE with 40 μm microspheres (Figure 1A) and 25.0±29.8 months since the diagnosis of HCC. Unadjusted median OS following DEB-TACE with 40 μm microspheres was 14.2±9.7 months and 17.2±15.8 months for patients classified with BCLC stages C and D, respectively. In multivariate Cox model regression analyses, a significant interaction was found that Okuda stage (P=0.03) and CLIP score (P=0.05) were significantly associated with OS after adjusting for other covariates (Figures 1A-1B). No other predictive factors were found in these analyses (Table 6).

**Table 6:** Multivariate Cox regression for overall survival* Abbreviations: BCLC, Barcelona-Clinic Liver Cancer; ECOG, Status Eastern Cooperative Oncology Group; MELD: Model for End-Stage Liver Disease; CLIP, Cancer of the Liver Italian Program *From the date of first DEB-TACE treatment to the date of death due to any cause or the end of the observation period

Covariate	P-value
Age	0.8145
Lesion size	0.7043
BCLC stage (D vs. C)	0.5310
ECOG status	0.9877
MELD score	0.2561
Okuda stage (II and III vs. I)	0.0297
CLIP score	0.0399
Milan status (Yes vs. No)	0.6816

**Figure 2:**
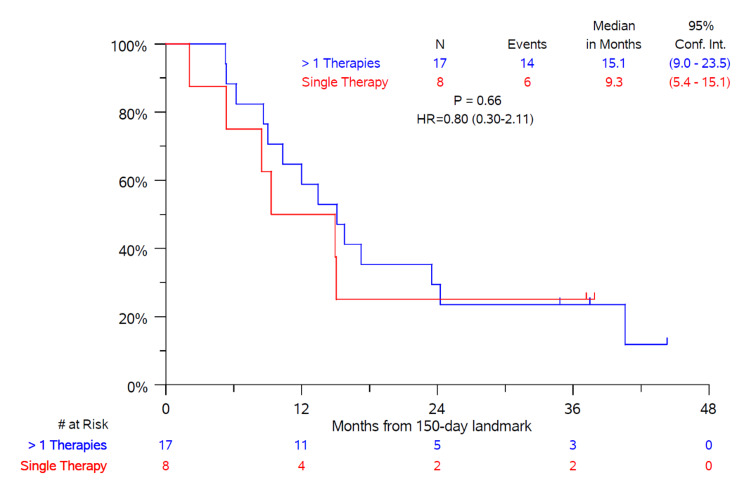
Overall survival landmark analysis comparing patients receiving a single DEB-TACE treatment with 40 μm drug-eluting microspheres loaded with 75 mg of doxorubicin versus multiple DEB-TACE treatments and/or undergoing microwave ablation Only patients surviving at least 150 days* (landmark) after the first DEB-TACE treatment were included in the analysis. *mean time between treatments for patients receiving multiple treatments with 40 μm drug-eluting microsphere loaded with 75 mg of doxorubicin Abbreviations: DEB-TACE: drug-eluting bead transarterial chemoembolization

A landmark analysis comparing subjects receiving a single DEB-TACE treatment versus those receiving multiple DEB-TACE treatments or MWA showed no significant difference between the two groups (Figure 2). Only patients surviving at least 150 days following the first DEB-TACE treatment were included in this analysis since this represented the mean time period between treatments for those receiving multiple DEB-TACE treatments with or without also undergoing MWA.

## Discussion

The present study demonstrated that DEB-TACE using a small-sized, drug-loaded microsphere for the treatment of high-risk, unresectable HCC patients is a safe and effective option for patients with BCLC stages C and D who do not have extrahepatic involvement. While the majority of publications describing the use of DEB-TACE for the treatment of HCC have focused on its use in lower-risk BCLC stages A and B patients, many patients present with locally advanced disease at the time of diagnosis and are not suitable candidates for curative treatments such a surgical resection. As a result, palliative treatments that have acceptable safety profiles that can provide the potential for favorable treatment response and prolonged survival are needed.

The BCLC staging system is useful for providing an outline for treating unresectable HCC because of its ability to classify patients with advanced liver disease and compromised performance status based on the severity of liver disease rather than the overall tumor burden. Unfortunately, the use of the latter can result in the incorrect assumption that these patients have a terminal disease [24]. While there is no consensus as to which staging system is best for predicting the survival of patients with HCC, several medical societies recommend the use of the BCLC system for patients with advanced HCC who are not candidates for surgery [5-6,25]. The BCLC system has been validated to predict survival for patients at differing stages based on the treatment approach.

Results from the present study suggest that the outcomes of the Okuda classification system and CLIP system are predictive of survival in BCLC stage C and D patients. The Okuda staging system was the first classification system to combine tumor sizes, measures of liver function, bilirubin levels, serum albumin, and tumor size with patients classified as either stage I (not advanced), stage II (moderately advanced), or stage III (very advanced) [18]. The Okuda system has been shown to be more predictable in patients with advanced HCC compared to patients diagnosed at earlier stages [26]. The results of the present study confirm the utility of the Okuda system for patients with advanced HCC.

The CLIP score takes into account the patient’s Child-Pugh status, tumor characteristics, including tumor morphology, whether the patient has diffuse or massive disease, AFP levels (<400 or >400 ng/dL), and whether portal vein thrombosis is present or not [19]. The scores associated with the above parameters are totaled and then patients are classified into groups (0 to 6) based on the sum of these scores. The cause of underlying disease, the presence of extrahepatic metastases, and performance status are not included in the scoring system. Since patients with extrahepatic involvement were not included in the present study, it is not surprising that the CLIP score was predictive of patient outcomes. The use of the CLIP scoring system has been shown to be more accurate than the Okuda classification system for identifying patients with a good prognosis and has advantages for use in patients with a poorer prognosis [26].

Since the present study was based on treatment in a “real-world” setting, approximately a third of the patients had previously undergone one or more TACE, DEB-TACE, or MWA procedures. Only two patients in this series had previously received systemic therapy with the tyrosine kinase inhibitor sorafenib. The use of sorafenib is limited in our patent population with unresected HCC as a result of the limited improvement in OS associated with sorafenib in these patients, the high rate of adverse events, the potential for life-threatening complications, and the frequent need to reduce dosage or discontinue therapy as a result of drug-induced side effects [27-28].

The use of a smaller-sized doxorubicin-loaded microsphere was selected for patients in this study due to the possible benefits of ensuring more distal embolization as a result of the smaller particles being able to penetrate deeper into the targeted tumor to deliver the chemotherapeutic drug [28]. The present study had similar findings to studies reporting on the use of smaller diameter beads in patients with HCC where there was an increase in extrahepatic toxicity and hepatobiliary injury [29-30]. The percentage of patients developing postembolization syndrome within 30 days of initial DEB-TACE with the 40 μm beads in the present study was low and occurred at the same frequency reported by Aal et al. (19%) in association with the use of 75 μm beads. There was also a low rate of significant lab-related toxicities during the 90-day period following DEB-TACE. There were four deaths that occurred during the first month following DEB-TACE. This reflects the fragile condition of these patients, with extensive HCC burden and other comorbidities. One patient died following a fall, which was likely due in part to the presence of multiple brain metastases observed in imaging studies after their fall. While being an exclusion factor for this study, the presence of metastatic disease was detected later in the course of treatment and was a significant cause of morbidity for this patient.

Overall survival is recommended for use as a primary endpoint for cancer treatment studies since it is both objective and clinically relevant [31]. The median OS of 15.0 months for patients in the current study compares favorably to an earlier study by Lewandowski et al. where they report a much lower median OS of 6.3 months associated with conventional oil-based TACE in 172 BCLC stage C patients [32]. Kalva et al. reported a median OS of 13.3 months for DEB-TACE using 100-300 μm beads loaded with 50 mg of doxorubicin in 80 BCLC stage C patients [33]. These findings align with those of the present study. More recently, Ray et al. reported a median OS of 19.9 months associated with the use of larger 100-300 μm or 300-500 μm beads in patients with BCLC stage C and D HCC. Of note is the different ratio of stages C and D patients in the study by Ray et al. (70% stage C and 30% stage D) [24] compared to the present study (28% stage C and 72% stage D).

While some define BCLC stage D as “terminal” cancer with the goal of providing palliative or supportive care [34], the results of the present study demonstrate the ability to prolong the life of high-risk patients using local regional therapy while avoiding systemic therapies like sorafenib. Of note is that four of the five patients still alive at the end of the study period were classified as BCLC stage D, and these patients had a mean average survival of 43.3 months (range 40.3 to 50.0 months) from initial DEB-TACE.

There are several potential limitations associated with the present study. The non-randomized, retrospective study design and the use of data from a single center suggest a potential for treatment bias. The small number of patients and the inclusion of 14 patients who had previous DEB-TACE or MWA treatments are also problematic. While the above limits the ability to make definitive conclusions from the results of the present study, the results provide interesting insights into the potential for using local regional therapy for patients with HCC who previously may have been judged to have too advanced a disease to be considered for treatment. Further clinical studies are warranted to explore this further.

## Conclusions

The present real-world study confirms that DEB-TACE using 40 μm doxorubicin-loaded microspheres is safe and effective for the treatment of unresectable patients with BCLC stages C and D advanced HCC. Select patients experienced both tumor response and prolonged OS. Okuda stage and CLIP score independently predicted OS after adjusting for other covariates. There was no difference in outcomes between patients treated with DEB-TACE with or without MWA. These results suggest that the use of smaller-sized doxorubicin-loaded microspheres, which enables more distal embolization and deeper penetration of localized chemotherapy into the targeted tumor, can successfully treat advanced HCC. Patients with advanced, high-risk unresectable HCC should be considered for DEB-TACE as opposed to supportive or palliative care.
